# Astrocytes: Role in pathogenesis and effect of commonly misused drugs in the HIV infected brain

**DOI:** 10.1016/j.crneur.2023.100108

**Published:** 2023-08-29

**Authors:** Jessalyn Pla-Tenorio, Angela M. Roig, Paulina A. García-Cesaní, Luis A. Santiago, Marian T. Sepulveda-Orengo, Richard J. Noel

**Affiliations:** a Seattle Children's Hospital, MS OC.7.830, 4800 Sand Point Way NE, Seattle, WA, 98105-0371, United States; b Bella Vista Hospital, Family Medicine Residency, Carr. 349 Km 2.7, Cerro Las Mesas, Mayaguez, PR, 00681, Puerto Rico; c Ponce Health Sciences University, School of Medicine, Department of Basic Sciences, 395 Industrial Reparada, Zona 2, Ponce, PR, 00716, Puerto Rico

**Keywords:** Astrocyte, HIV, Substance use disorder, Neurotransmitter

## Abstract

The roles of astrocytes as reservoirs and producers of a subset of viral proteins in the HIV infected brain have been studied extensively as a key to understanding HIV-associated neurocognitive disorders (HAND). However, their comprehensive role in the context of intersecting substance use and neurocircuitry of the reward pathway and HAND has yet to be fully explained. Use of methamphetamines, cocaine, or opioids in the context of HIV infection have been shown to lead to a faster progression of HAND. Glutamatergic, dopaminergic, and GABAergic systems are implicated in the development of HAND-induced cognitive impairments. A thorough review of scientific literature exploring the variety of mechanisms in which these drugs exert their effects on the HIV brain and astrocytes has revealed marked areas of convergence in overexcitation leading to increased drug-seeking behavior, inflammation, apoptosis, and irreversible neurotoxicity. The present review investigates astrocytes, the neural pathways, and mechanisms of drug disruption that ultimately play a larger holistic role in terms of HIV progression and drug use. There are opportunities for future research, therapeutic intervention, and preventive strategies to diminish HAND in the subset population of patients with HIV and substance use disorder.

## Highlights

• Astrocytes are critical reservoirs of HIV neurotoxins that contribute to NeuroHIV.
• Illicit substance use disrupts the normal neurotransmitter homeostasis by astrocytes.
• The overlap of effects by HIV neurotoxins and illicit drugs offers areas for future research.

## Graphical abstract

**Figure:**
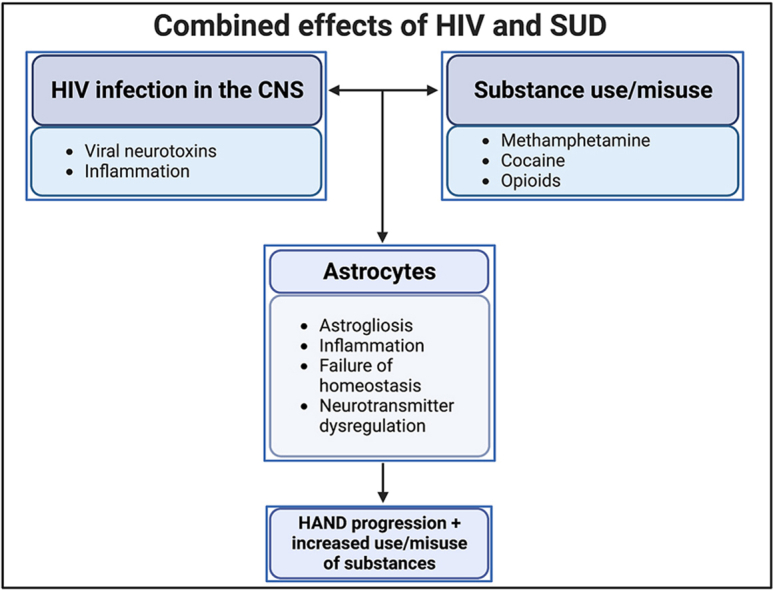

## 1 Introduction

The HIV and drug misuse epidemics are interrelated health and social challenges we face today in the United States (Kim et al., 2018a). Intravenous (IV) drug use is the second leading cause of HIV infections in the United States and accounts for 10% of new infections (Prevention and C.f.D.C.a., 2021). Substance misuse, both IV and non-injection, often increases high-risk behaviors, such as unprotected sex, multiple partners, and needle sharing, that augment the chances of exposure to HIV (Wang and Maher, 2019). Approximately 17–53% of HIV patients receiving care are prescribed opioid prescriptions to treat their chronic pain; with an estimated projection of 2–65% reporting misuse based on surveillance system data analysis (Ventuneac et al., 2021; Lemons et al., 2019). In addition, the pathologies of HIV infection and substance use intersect in the brain, impairing cognitive capacity and mental health (Tyagi et al., 2016).

Within weeks of initial HIV infection, infected monocytes cross the blood brain barrier (BBB) in response to chemokines and produce neuroinflammation that persists despite undetectable levels of viremia maintained through antiretroviral therapy (ART) (Jaureguiberry-Bravo et al., 2018). This persistent inflammatory state drives cumulative damage to the brain. In approximately 50% of those infected, this inflammatory state leads to a series of cognitive and neuropsychological impairments, referred to as HIV-associated neurocognitive disorders (HAND). Despite the control of replication via ART, latent HIV infection of glial cells leads to the production of viral gene products, particularly the early proteins Tat and Nef, implicated in inflammation and neurotoxicity in the central nervous system (CNS) (Chompre et al., 2013; Sami Saribas et al., 2017; Rivera et al., 2019; Cheng et al., 1998). This phenomenon contributes to HAND progression.

Astrocytes are the majority glial cell type in the brain and play diverse roles in the development and function of the brain; they are vital in formation of the BBB, and for the maintenance of proper electrolyte concentrations, reuptake, and metabolism of neurotransmitters from the synaptic clefts (Rodriguez et al., 2019). Impairment of any of these functions leads to disruption of homeostasis and the subsequent dysfunction of the CNS (Rodriguez et al., 2019).

Cognitive impairments caused by HIV arise even in patients with undetectable viremia during ART. HAND can be sub-classified, ranging from severe HIV-associated dementia (HAD) to milder forms like mild neurocognitive disorders (MND) and asymptomatic neurocognitive impairments (ANI) (Antinori et al., 2007). Since the implementation of antiretroviral therapy, the prevalence of HAD has diminished, but the prevalence of the milder forms of cognitive impairment, MND and ANI, has remained the same (Saylor et al., 2016). Even under ART, around 25% of HIV patients develop at least one neurologic symptom such as sensory, cognitive or motor impairment (Vivithanaporn et al., 2010).

Past studies have shown that the use of such as methamphetamine, cocaine, or opioids in the context of HIV infection contributes to severe neuroinflammation and development of HAND (Tyagi et al., 2016; Yu et al., 2017; Festa and Meucci, 2012). Through a variety of mechanisms, these substances exacerbate inflammatory responses within the CNS, leading to severe neurotoxicity and eventually cognitive decline. The cohort of HIV patients suffer from a substance use disorder exhibits a faster progression of cognitive decline when compared to HIV patients who do not (Tyagi et al., 2016; Yu et al., 2017; Festa and Meucci, 2012).

The incidence of HAND remains high among some individual patients who are ART adherent and maintain low viral loads. The added comorbidity of substance use disorder (SUD) may represent an additional risk factor for HAND progression. Studies investigating the intersection between HIV and drug use have continued to arise throughout the years, delineating the combined effects that HIV and drugs have on neuroinflammation and exacerbation of HAND. HIV + patients show cortical thinning in the prefrontal cortex (PFC), an area that is also affected in the presence of cocaine which appears hypoactive. Other studies have shown reduced activity of the PFC and worse cognitive function in rats exposed of HIV-1 Tat and cocaine (Wayman et al., 2015; McIntosh et al., 2022). Below, we focus on several areas of overlapping inflammation and toxicity caused by HIV and some selected drugs, with an emphasis on the roles of astrocytes and how HAND is worsened. We will attempt to further define the link between HIV astrocytic infection in the brain, its pathological effects, and the augmented severity of neuroinflammation caused by the concomitant use of select drugs that leads to worsening of HAND.

## 2 Astrocytes as HIV reservoirs

The elaborate nature of HIV-1 infection in the CNS has generated wide debate over site of viral replication and the role of astrocytes, the most abundant cells in the brain. With a myriad of functions in both physiological and pathological settings, these glial cells provide a vast communication network in the CNS by means of signaling proteins, cell-to-cell interactions, and intercellular signaling (Malik et al., 2021). Although HIV-1 replication in the CNS occurs mostly in perivascular macrophages and microglia where productive CNS dissemination has been found in vivo (Borrajo et al., 2021a; Woodburn et al., 2022), in vitro astrocyte infection by HIV-1 has been inefficient with persistent infection of as few as 0.5% of cells (Li et al., 2020; Edara et al., 2020). This refractoriness may be due in part to lack of sufficient CD4 expression, however, in vitro use of cell free HIV stocks that only contain mature viral particles further impede transmission. In cell-to-cell models, CXCR4 binding to immature HIV gp120 can produce astrocyte infection even when kept physically apart from HIV infected lymphocytes (Li et al., 2020; Valdebenito et al., 2021), although the exact mechanism remains unclear. Similarly, Luo and colleagues confirmed viral infection of astrocytes by way of dose-dependent cell-to-cell contact with HIV-infected CD4^+^ T cells (Luo and He, 2015). While most studies suggest astrocyte infection to be restricted or non-productive (Li et al., 2020) where astrocytes endocytose cell-free virus and have it compartmentalized to later be released (Russell et al., 2017), Edara, et al., were able to differentiate between astrocytes with an active or a latent/restricted infection by presence of an active or silent viral promoter, respectively. They propose that these few active astrocytes that are inducible to reactivation are responsible for limited productive infection found in vitro which correlates with Valdebenito's proposed astrocyte subpopulations (Edara et al., 2020; Valdebenito et al., 2021). A more recent study comprehensively showed that HIV within astrocytes has the potential to reseed peripheral target cells in humanized rodent models, implicating the importance of eradication strategies targeted toward astrocytic reservoirs (Valdebenito et al., 2021; Lutgen et al., 2020).

Despite controversies regarding restrictions on infection and replication in astrocytes, once viral entry has been achieved, HIV-1 is integrated as a stable provirus (Malik et al., 2021; Borrajo et al., 2021a) and regardless of promoter activity, presence of the provirus induces infected phenotype associated with dysfunction and neurotoxicity (Edara et al., 2020). For the purpose of this review, we will highlight models of astrocytes as HIV reservoirs given that astrocyte make up about 60% of cells in the CNS and even conservative infection of about 1–3% found in vivo and in vitro would produce significant alterations to homeostasis (Valdebenito et al., 2021). Rather than a source of replicating virus, HIV-infected astrocytes produce modest levels of a subset of HIV proteins including Tat and Nef (Edara et al., 2020). It is generally known that HIV triggers astrocytic activation and release of neuroactive gliotransmitters (Woodburn et al., 2022; Ton and Xiong, 2013; Cisneros and Ghorpade, 2012) which modulate the homeostatic conditions and functions of neighboring cells through protective mechanisms to limit the spread of infection and/or noxious stimuli (Borgmann and Ghorpade, 2015; Sofroniew and Vinters, 2010). In the context of chronic neuroinflammation, astrocyte function is further impaired (Valdebenito et al., 2021) affecting essential and interactive roles in synaptic transmission as part of the tripartite synapse (Borgmann and Ghorpade, 2015). Astrocytes cover about 90% of the BBB surface and modulate its permeability, therefore, disrupting astrocytic function will impact BBB integrity and overall brain homeostasis (Li et al., 2010; Mathiisen et al., 2010; Galea, 2021). Accumulating evidence suggests that astrocytes are also involved in neuronal support through regulating neurotransmitter release and synaptic plasticity (Ota et al., 2013). Infected astrocytes have also been found to use intercellular channels to induce apoptosis in adjacent uninfected cells, promote cytotoxicity and sustain inflammation (Valdebenito et al., 2021; Ash et al., 2021). Consequently, astrocyte impairment will result in a negative impact on neuronal survival. Like microglia, astrocytes have roles in the phagocytic maintenance of the CNS and cytokine production and are involved in synaptic transmission, brain plasticity, and “neuroprotection” (Russell et al., 2017; Borgmann and Ghorpade, 2015; Narita et al., 2006; Corkrum et al., 2019; Qin et al., 2018). The reciprocal nature of astrocyte-microglia communications via cellular projections, secreted mediators and extracellular vesicles, provide the CNS’ robust response to neuronal injury in both physiological and pathological settings (Matejuk and Ransohoff, 2020). In the presence of HIV, these non-neural cells sustain changes in function and at the transcriptomic level, as evidenced by single-cell RNA-sequencing; resulting in impaired microglia with prolonged activation, aberrant myelination and cytokine release, all which support reactive astrogliosis, pathologic differentiation and apoptosis in astrocytes (Spurgat and Tang, 2022; Borrajo et al., 2021b).

Recognizing that there is considerable controversy regarding the infectivity of astrocytes and consequently, their role as HIV reservoirs, we suggest that astrocyte predominance as CNS resident cell is compelling to mitigate low infectivity rates. Although the determination of productivity vs latency in these infected astrocytes remains unknown, harboring provirus by itself triggers astrogliosis with the associated neurotoxicity and regulatory dysfunction. Furthermore, the lack of antiretrovirals preventing viral transcription, means there is no pharmacological block to production of early viral neurotoxins, Nef and Tat, in latent reservoirs.

## 3 Astrocytic dysfunction in the reward pathway BY HIV and drugs

Substance use disorder is a persistent challenge to HIV-infected patients’ healthcare and treatment adherence. Even with decades of research in the biology and psychology of addiction, the poor success of current treatments suggests we have much to discover regarding the underlying mechanisms involved in establishing addiction and SUD. In the case of SUD in the setting of HIV infection, even less has been elucidated on how the presence of HIV and viral proteins further alters these physiological changes.

Astrocytes are involved in the regulatory neural network of reward seeking-behavior, raising the possibility for therapeutic exploitation in combating addiction (Wang et al., 2022; Kang et al., 2020). Scofield and colleagues, for example, showed that astrocyte activation by designer receptors exclusively activated by designer drugs (DREADDs) increases glutamate levels in the NAc core, reducing cue-induced cocaine-seeking behavior (Scofield et al., 2015). Astrocyte activation and activity of the glutamate transporter (GLT-1) excite ventral tegmental area (VTA) GABAergic neurons, reducing the activity of the dopaminergic neurons and increasing avoidance behavior (Gomez et al., 2019; Koob and Volkow, 2016). Due to the role of astrocytes in stimulus-induced brain plasticity, astrocyte impairment by substance use, HIV infection, or the combination of the two, could lead to further alteration of neural connections and worsening of addiction relapse behaviors. Below, we explore the additive effects of HIV and SUD on astrocyte function within the reward pathway and the consequences of these maladaptations.

Substance use disorder may yield chronic, neurobiological perturbations alone and, in HIV-infected individuals, can lead to faster development of HAND (Nath et al., 2008; Bouwman et al., 1998). Repeated use of drugs alters neurotransmitters from glutamatergic, dopaminergic, and GABAergic neurons, as similarly seen in chronic HIV infection (Illenberger et al., 2020). These neurotransmitters are found in the reward pathway, including the VTA, NAc, the prefrontal cortex (PFC), and the amygdala, which are engaged in the behavioral response to addictive substances (Pariyadath et al., 2016; Volkow et al., 2019). Furthermore, dysregulation of the glutamatergic, dopaminergic, and GABAergic systems is implicated in developing cognitive impairments, representing potential overlapping mechanisms of HAND and SUD. For example, in the setting of HIV, VTA signaling can be disrupted by Tat, the HIV transactivator protein, that augments reward cues for multiple drugs when acting alone. When coupled with exposure to methamphetamine, Tat modifies dopamine transmission and increases dopaminergic neurons in the VTA (Kesby et al., 2017).

All substances of misuse, including those discussed below, disrupt the dopaminergic system, and elevate CNS dopamine levels. Therefore, exploring the interactions of the dopaminergic system in substance dependence, HIV infection, and astrocytes is essential to fully understand the impact of substance misuse on astrocyte-mediated HIV pathology.

### 3.1 Dopaminergic system

The dopaminergic system has been widely studied independently in addiction and the development of HAND (Nath et al., 2008; Purohit et al., 2011, 2013). However, further research investigating the combination of HIV and SUD effects on the dopaminergic system is needed. Dopaminergic neurons project to brain regions throughout the reward circuitry. The PFC and striatal subregions have connections within the VTA. Within the PFC, dopaminergic neurons express D1 and D2-like receptors, which are G protein-coupled receptor (GPCR) families with distinct properties. D1-like receptors (D1 and D5) signal via stimulatory (Gαs) GPCRs resulting in the production of cAMP and PKA. On the other hand, D2-like receptors (D2, D3, and D4) signal via inhibitory (Gαi/o) GPCR and reduce adenylyl cyclase activity. These receptors are also present in glutamatergic pyramidal neurons, GABAergic interneurons, and astrocytes (Miyazaki et al., 2004), which can control the stimulation or inhibition of downstream activity throughout the system, modulating neural signaling (Miyazaki et al., 2004; Giacometti and Barker, 2019; Tseng and O'Donnell, 2007).

Decreased or increased dopamine levels in different cell types are a significant regulator of inflammation (Pacheco et al., 2014). Although different types of dopamine receptors, which range from DRD1-DRD5, are found to be expressed in adaptive and innate immune cells, including glial cells (Mastroeni et al., 2009; Farber et al., 2005), this review focuses primarily on the immune effects of dopamine on astrocytes as an impact of substances of misuse and HIV. For example, DRD3 is expressed in mouse, rat, and human astrocytes (Miyazaki et al., 2004; Huck et al., 2015; Elgueta et al., 2017; Kumar and Patel, 2007). DRD3 exacerbates neuroinflammation when increasingly expressed in astrocytes and has been found to inhibit cAMP production (Franz et al., 2015). However, other studies show that dopaminergic signaling in astrocytes can exert a protective effect on neurodegeneration (Yan et al., 2015; Shao et al., 2013). The lack of DRD2 in astrocytes can also contribute to neurodegeneration, as found in a study focused on demonstrating severe inflammation by glial cells in nigral dopaminergic neurons in DRD2 null (−/−) mice (Shao et al., 2013). Altogether, these findings suggest that astrocytic dopaminergic receptors are essential and have a critical function in modulating neuroinflammation and neurodegeneration.

Dopamine itself is known to play a major role in regulating immune functions. It has been found to be involved in cytokine modulation, phagocytosis, proliferation and chemotaxis of myeloid cells (Pinoli et al., 2017; Nickoloff-Bybel et al., 2020; Channer et al., 2022). Dopamine and DRD1 signaling have been shown to inhibit NLRP3 inflammasome activation and functions that are critical in controlling excessive inflammation as seen in inflammatory diseases (Yan et al., 2015). However, some studies have also demonstrated increases in inflammasomes and gene expression in HIV infected macrophages in treatment with cocaine (Yan et al., 2015). Although possibly conflicting on the mechanistic level, elevated dopamine levels and receptor signaling leads to dysregulation in inflammatory responses and safeguards. Additionally, previous studies have shown that the increased levels of dopamine concentration, as seen in substance use, increase HIV replication and infection in macrophages (Matt et al., 2021). Increased CCR5 expression, mediating the entry of HIV virions into myeloid cells, has been seen in non-human primates in the setting of methamphetamine and cocaine (Marcondes et al., 2010; Najera et al., 2016). Furthermore, a study performed on human derived macrophages from purified and matured PBMCs as well as inducible pluripotent stem-derived microglia demonstrated dopamine's ability to change the confirmation of CCR5 on macrophages and microglia (Matt et al., 2021). This study supports previous literature in establishing not only the immunomodulatory potential that high levels of dopamine exert on increasing HIV infectivity but also the implications towards CCR5 antiviral targeting therapy decreased efficacy with Maraviroc in HIV infected individuals who use substances. Although ART has been shown to decrease the neuropathogenicity displayed by HIV in the dopaminergic regions, substantial dysfunction, microglial activation, and inflammation persists (Nickoloff-Bybel et al., 2020; Vera et al., 2016).

In the setting of HIV infection, the dopaminergic system appears to be particularly vulnerable to exacerbated neurotoxicity from SUD. Dysregulation in dopamine levels in the ventral striatum and putamen is observed in individuals with severe HAND (HIV-associated dementia, HAD) (Wang et al., 2004). Patients with HAD show a decrease in dopamine transporter (DAT) expression in these structures, contributing to declining cognitive function and worsening SUD (Wang et al., 2004; Chang et al., 2008). Studies have shown a direct interaction of HIV-Tat with the DAT protein that decreases dopamine reuptake (Zhu et al., 2009; Wallace et al., 2006). Moreover, alterations in dopamine receptors of the PFC are associated with HIV (Gelman et al., 2012). A study investigating Tat-expressing transgenic mice demonstrated hyperexcitability and dendritic damage secondary to a reduction in striatal dopamine D2 receptors in medium spiny neurons (MSNs) (Gelman et al., 2012; Schier et al., 2017). In particular, NAc MSNs play a role in motivational and rewarding behavior (Klawonn and Malenka, 2018). Studies have reported morphological alterations in female HIV-1 transgenic rats, increasing stubby spines (Roscoe et al., 2014). Stubby spines diminish neuroplasticity by reducing contact between neurons (Javadi-Paydar et al., 2017). Since MSNs receive regulatory input from the PFC and VTA, altering their morphology can decrease dopaminergic synapses and other synaptic contacts (Zahm and Brog, 1992; Nestler, 2005; Schmidt et al., 2009).

**Fig. 1:**
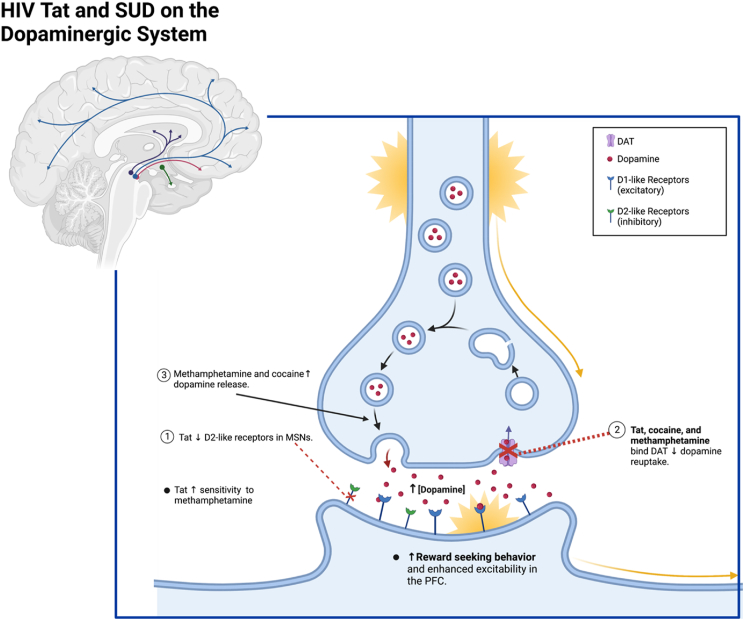
HIV neurotoxin Tat and SUD effects on the Dopaminergic System.

Other proteins, such as dopamine transporter (DAT) and vesicular monoamine transporter 2 (VMAT2) regulate the effects of dopamine (Jones et al., 1995; Amara and Sonders, 1998). In contrast to the reduction of D2 signaling by Tat, the psychostimulant drugs cocaine and methamphetamine prolong dopamine action in the synapse by blocking presynaptic reuptake by monoamine transporters, including DAT and VMAT2 (Jones et al., 1995; Amara and Sonders, 1998; Ritz et al., 1987; Han and Gu, 2006). These same drugs potentiate dopamine release from presynaptic neurons from the striatum resulting in a reward stimulus. This enhancement subsequently leads to a lower reward threshold (Kesby et al., 2016). The findings from this study suggested that exposure to Tat can enhance the sensitivity to methamphetamine-induced reward (Kesby et al., 2016). A study investigating motivational states in an HIV-1 Tat transgenic mouse model treated with cocaine reported decreased performance on tasks involving frontal-subcortical circuitry and reduced NAc volume. These alterations were attributed to the dysregulation in the dopaminergic system caused by combined interactions of cocaine and Tat (Bertrand et al., 2015). HIV-1 transgenic rats express most proteins of HIV, including Tat, and exhibit a reinforcing effect in cocaine self-administration and enhanced excitability in the PFC (Wayman et al., 2016; McIntosh et al., 2015). Additionally, the rewarding effects of alcohol and cocaine are augmented in the presence of Tat in the CNS in a conditioned place preference paradigm (Paris et al., 2014; McLaughlin et al., 2014). On the other hand, HIV-Tg rats exhibited disruption in choice behavior preference and a reduced shift in preference from sucrose to cocaine compared to their non-Tg Fisher344 counterparts (Bertrand et al., 2018). The HIV-Tg rats depicted a similar reinforcement for sucrose-seeking behavior and showed decreased dopamine transporter function in the striatum. This provides a basis for how HIV may alter the dopaminergic system and homeostasis even prior to cocaine exposure (Bertrand et al., 2018). Furthermore, infusions of gp120 also decrease dopamine function, indicating that various HIV-1 proteins may enhance the rewarding effects of drugs of abuse and reduce dopaminergic function (Kesby et al., 2016; Bansal et al., 2000). Taken together, the aforementioned mechanisms focused on the highly studied HIV neurotoxin Tat (summarized in Fig. 1) contribute to decreased dopamine turnover, increased levels of extracellular dopamine, and enhanced reward-seeking behaviors.

Dopaminergic neurons found throughout the reward circuitry, with high concentration in the PFC and striatal subregions with connections to the VTA, express both D1(excitatory) and D2-like (inhibitory) receptors. Dysregulation in dopamine levels observed in HAND are due to decrease in dopamine transporter expression, resulting in higher baselines of dopamine available in the synaptic cleft. HIV-1 Tat causes hyperexcitability and dendritic damage secondary to a reduction in striatal dopamine D2 receptors in medium spiny neurons (MSNs) (1). Tat can also bind to DAT, inhibit its function, and reduce dopamine reuptake. (2) Use of psychostimulant drugs methamphetamine and cocaine block dopamine reuptake, increasing dopamine in the synaptic cleft. (2) In addition, meth can potentiate dopamine release from presynaptic neurons from the striatum. (3) This results in enhanced excitability to the postsynaptic neurons causing a reward stimulus.

### 3.2 Glutamatergic system

Glutamate is the primary excitatory amino acid in the CNS and plays a crucial role in synaptic transmission and function. Since astrocytes communicate bidirectionally with neurons through the glutamate-glutamine cycle in what is known as the tripartite synapse, disrupting glutamate homeostasis would result in a direct influence on neuronal presynaptic release and postsynaptic responsiveness (Wang et al., 2022). Accumulating glutamate in the synaptic cleft can trigger long-term potentiation via AMPA receptors located in the postsynaptic neurons. However, excessive synaptic glutamate has proven to lead to excitotoxicity by activating NMDA receptors in the postsynaptic membrane, consequently causing dendritic damage and even neuronal death in various brain areas (Chen et al., 2020a). Within astrocytes found in the NAc, activation of the mu-opioid receptors led to the elevation of cytoplasmic calcium levels and subsequent gliotransmitter glutamate release, resulting in NMDA receptor activation (Corkrum et al., 2019). Furthermore, the astrocytes of the VTA, unique in morphology, gene expression, and physiology, channel diverse input to act in approach and avoidance learning primarily through the activation of glutamate transporters: GLAST (EAAT-1) and GLT-1 (EAAT-2), whose roles are to regulate extracellular glutamate levels in the brain (Gomez et al., 2019; Koob and Volkow, 2016; Xin et al., 2019).

HAND is associated with elevated extracellular glutamate levels and overall disrupted glutamate homeostasis in the CNS (Saylor et al., 2016; Bairwa et al., 2016; Cassol et al., 2014; Ernst et al., 2010; Ferrarese et al., 2001; Potter et al., 2013; Sailasuta et al., 2009; Schifitto et al., 2007; Stankoff et al., 2001; Vazquez-Santiago et al., 2014). Clinical studies detected glutamate in the cerebrospinal fluid of HAND patients, correlating with cognitive impairment levels (Cassol et al., 2014; Ferrarese et al., 2001). When HIV is present in the CNS, virus-infected microglia and astrocytes release cytokines and chemokines, causing neuronal inflammation. One of the released chemokines, CCL2, has been shown to enhance NMDAR-mediated excitotoxicity in the CA1 hippocampal zone by decreasing the high-affinity expression of glutamate transporters (Chen et al., 2020a). HIV-1 Tat is one of the viral proteins that mediates increased glutamate in the CNS. Tat can also activate NMDA receptors in the cortex and hippocampus of rats (Haughey et al., 2001; Song et al., 2003; Aksenov et al., 2012). A study on rat cortical neurons demonstrated potentiated excitotoxicity due to Tat exposure, subsequent neuroinflammation, and even cell death (Haughey et al., 2001). Tat also mediates neurotoxicity through glutamate release acting on AMPA receptors (Fitting et al., 2014). The upregulation of AMPARs and NMDARs can lead to dendritic shortening, which is prevalent in HIV-1 infected individuals, linking memory deficits seen in cognitive decline (Mattson et al., 2005). Coexposure of Tat with opiates exacerbates HIV-1 induced synaptodendritic degeneration (Fitting et al., 2010a, 2014). Morphine, via its action on mu-opioid receptors (MOR), causes calcium (Ca2+) release and mitochondrial destabilization in dendrites, further heightening the excitotoxic effect produced by Tat in NMDAR signaling. Moreover, Tat can stimulate IP3-mediated release of calcium (Berridge, 1987), which is enhanced by the addition of morphine. Tat and morphine have overlapping effects that can disrupt Ca2+ homeostasis and exacerbate neuronal injury (Fitting et al., 2014). In short, we can deduce that the various mechanisms of disrupted glutaminergic signaling, as described above, that are potentiated by substance use in the HIV infected brain contribute to the progressive neurocognitive decline frequently observed in this population.

Elevated extracellular glutamate levels in the PFC and the NAc are also associated with drug-induced seeking behavior (McFarland et al., 2003; Scofield et al., 2016). Methamphetamine use and HIV-1 induce activation of astrocytes and have been demonstrated to decrease glutamate transporter mRNA levels, specifically EAAT-2, in primary human astrocytes, indirectly affecting the function of glutamate receptors located in neurons (Cisneros and Ghorpade, 2012). Cocaine alone can also decrease levels of GLT-1 and system xc-, a cysteine glutamate transporter in charge of regulating levels of extracellular glutamate, specifically in the NAc (Baker et al., 2002, 2003) in rats after self-administration. Downregulation of these transporters can cause glutamate spillover in the NAc core and is suggested to enhance the reinstatement of drug-seeking behavior for different addictive substances such as cocaine, methamphetamine, heroin, nicotine, and alcohol (Buch et al., 2012). A study using intra-NAc microinjections of TFB-TBOA, a selective astrocytic EAAT 1/2 inhibitor, in male and female Long-Evans rats reduced cocaine self-administration due to elevation of extracellular glutamate in the NAc through downregulation of GLT-1, specifically, but no effect in dopamine levels. These effects were not observed in areas like the VTA, ventral pallidum, or dorsal striatum. They also found that cocaine self-administration altered GluN2B-NMDA subunit expression in the NAc of rats, increasing the expression of this subunit in dopaminoceptive neurons that express DARPP-32 with a history of cocaine use (Yang et al., 2022). This data suggests the importance of glutamate in SUD and its function in the CNS.

**Fig. 2:**
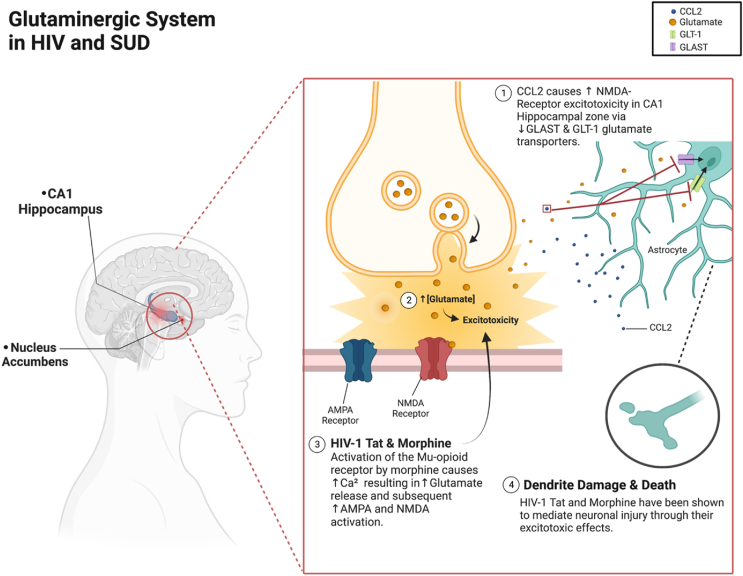
HIV and SUD effects on the Glutaminergic System.

Glutamatergic projections from the PFC to the NAc core are associated with the formation of addictive related behaviors (Li et al., 1999; McFarland and Kalivas, 2001; Pierce et al., 1998). In terms of HIV, Bowers and colleagues infused rats with a peptide containing Gia binding domain (GPR) to the cell permeability domain of HIV-Tat (Tat-GPR) in the PFC, which resulted in an enhanced reinstatement of cocaine-seeking behavior that correlated with an increase in extracellular glutamate in the NAc core (Bowers et al., 2004). This study suggests that HIV-1 Tat can potentially reinforce relapse to cocaine. However, there is still no conclusion on whether HIV has a synergistic behavior effect that could impact drug-seeking behavior through glutamatergic signaling. Fig. 2 summarizes the findings in this section and how the glutamate cycle could be involved in the potentiation of HAND and drug-seeking behavior.

Glutamate transporters GLAST and GLT-1(EAAT-1 and EAAT-2 in humans respectively), responsible for removing excess glutamate from the synaptic cleft, are downregulated by chemokine CCL2 released from HIV infected astrocytes. Similarly, methamphetamine use in the context of HIV-1 has been demonstrated to decrease EAAT-1 as well. (1) This results in enhanced NMDAR-mediated excitotoxicity in the CA1 hippocampal region contributing to memory and cognitive deficits seen in HAND. (2) Activation of Mu opioid receptors on astrocytes found in the NAc, leads to the elevation of cytoplasmic calcium levels and glutamate release, further potentiating NMDA receptor activation. (3) Tat and morphine together have displayed this Ca2+ homeostasis disruption and have been seen to exacerbate neuronal injury (4) through the increased glutamate activating both NMDA and AMPA receptors.

### 3.3 GABAERGIC system

As mentioned above, all substances of misuse alter the dopamine circuitry in the CNS including, especially excitatory drugs. Moreover, studies using animal and human models have shown that increasing GABA concentration can reverse the effects of dopamine in the brain (Silverman, 2018). Gamma-aminobutyric acid (GABA) is an inhibitory gliotransmitter secreted by glial cells. GABA receptors are divided into GABA A receptors and GABA B receptors. GABA A receptors are fast-acting regulatory integral membrane ion channels sensitive to chloride and HCO3 anions (Doyon et al., 2016; Rahman et al., 2022). In contrast, GABA B receptors are slow-acting G-protein coupled receptors (Rahman et al., 2022). Vigabatrin, a drug responsible for inhibiting the enzyme for GABA catabolism (GABA-AT), has been found to prevent cocaine addiction in rat and baboon models (Silverman, 2018; Dewey et al., 1998; Kushner et al., 1999; Brodie et al., 2003) and has had positive outcomes in nicotine, methamphetamine, alcohol, and heroin addiction studies (Dewey et al., 1999; Gerasimov et al., 1999). However, large doses of the drug, already used as a treatment for epilepsy, are needed for GABA-AT to have an effect on the brain, which, over an extended period, can cause retinal damage in patients (Silverman, 2018; Wild et al., 2009).

HIV can also cause disruption of inhibitory pathways, including the GABAergic system. Several studies have demonstrated increased GABAergic signaling and decreased excitatory synapses in hippocampal neurons caused by Tat protein, potentiating inhibitory neurotransmission (Hargus and Thayer, 2013; Shin and Thayer, 2013; Kim et al., 2008). Interestingly, studies have observed the opposite occurring in the cortex and striatum of mice, rats, and humans, increasing cortical excitation activities instead of inhibitory potentiation (Musante et al., 2010; Xu et al., 2016a, 2016b). Therefore, GABAergic interneurons are also targeted by HIV-1 proteins and SUD, making the infected brain vulnerable to hyperexcitation. Accumulating evidence has revealed damage of GABAergic interneurons caused by Tat and HIV-1 infection in post-mortem brain tissue and HIV mouse models (Buzhdygan et al., 2016; Fitting et al., 2013; Marks et al., 2016). However, this hyperexcitation does not seem to be governed by neuronal loss but by the loss of GABAergic markers such as GAD1, GAD2, and GJD2. A study found that, even with a history of drug use and ART, autopsy samples from HIV-infected patients showed a significant loss of these markers (Buzhdygan et al., 2016). The neuronal K–Cl cotransporter, KCC2, has been shown to disrupt GABAergic functioning in various neurological disorders, including HAND. HIV in the presence of morphine has been found to dysregulate KCC2 activity and decrease GABAAR-mediated hyperpolarization and inhibition (Barbour et al., 2020). KCC2 loss can lead to CCR5 activation, which may contribute to this underlying hyperexcitability. However, KCC2 expression and, subsequently, GABAAR function were rescued by a CCR5 antagonist in gp120 and opioids exposed neurons (Barbour et al., 2020). Briefly, this hyperexcitable state, mediated partly by KCC2 dysregulation, leads to neuronal damage and is a key characteristic of an HIV and morphine exposed brain.

Past studies have demonstrated how neurodegeneration can result from an imbalance in excitatory and inhibitory neural processes. Still, compared with dopamine, studies on glutamate and GABA have received poor attention in the context of HIV and SUD over the years, even with their high importance in these fields. Overall, the studies mentioned above suggest that the lack of GABAergic signaling in neurons results in a vulnerable brain prone to neuronal damage in neurodegenerative disorders. However, very little is known about how astrocytes regulate GABA-mediated signaling during HIV and SUD. The following sections will focus on significant classes of drugs commonly used among HIV-positive individuals and their direct effects on astrocytes and the neurotransmitter systems in the CNS.

## 4 HIV and drug effects on astrocytes and neuropathogenesis

### 4.1 Methamphetamine

The use of methamphetamines (meth) is associated with a 1.5-fold increased risk of HIV infection and is a widely used stimulant with over 1.6 million users in the United States (Chilunda et al., 2019; Plankey et al., 2007). The use of meth alone causes cellular changes in the CNS that lead to altered cognitive function (Chen et al., 2020b; Pastuzyn et al., 2012; Daberkow et al., 2008). Meth works by increasing the half-life of dopamine in the synaptic cleft through a variety of mechanisms. It blocks the reuptake of dopamine through the inhibition of the dopamine transporter, DAT, which under normal conditions transports the neurotransmitter into the presynaptic neuron or astrocyte to recycle dopamine for future use (Chilunda et al., 2019). Additionally, meth has a direct effect increasing vesicular release of dopamine through modulation of VMAT-2, a transport protein that loads dopamine into presynaptic vesicles for subsequent release (Chilunda et al., 2019). These increased levels of dopamine in the synaptic cleft lead to excitotoxicity and neuronal damage. Astrocytes aid in the recycling of dopamine and other neurotransmitters. Although astrocytic roles in the process of addiction have yet to be fully understood, we will focus on studies that have shed light on the use of meth in HIV infected astrocytes and the proposed neuroinflammation and degeneration seen to exacerbate the development of HAND.

In the context of HIV and meth use, astrocytes become senescent, resulting in toxicity to the neighboring neurons and glia through altered secretion of cytokines and chemokines. This alteration exhibited by senescent cells has been shown, through in vitro and animal models, to induce inflammation leading to neurodegeneration and cognitive impairment (Yu et al., 2017). Meth use in combination with HIV has been observed to induce mitochondrial dysfunction in human astrocytes through distinct mechanisms (Borgmann and Ghorpade, 2018). Chronic exposure to meth and HIV augments oxidative stress and ATP levels in astrocytes (Borgmann and Ghorpade, 2018). Specifically, HIV gp120 alongside meth, has been shown to increase oxidative stress and decrease mitochondrial function, provoking autophagy in SVGA astrocytes. Extrapolating these in vitro and in vivo findings, suggests that astrocyte damage will cause both a failure to restore homeostasis as well as induce apoptosis (Borgmann and Ghorpade, 2018). A finding by Park et al. demonstrated that meth on an EcoHIV model affected GFAP protein levels when compared to either treatment alone in the caudate putamen of mice brain (Park et al., 2021). Therefore, activation of astrocytes in the brain is one of the trademarks of enhanced neurotoxicity induced by combined exposure of meth and HIV (Cisneros and Ghorpade, 2012; Park et al., 2021). Yu and colleagues demonstrated that HIV and meth worked additively to further downregulate the Wnt/β-catenin pathway to mediate senescence and amplify the neurodegeneration (Yu et al., 2017). Besides disruption of this pathway, the combination of HIV gp120 and methamphetamine, in transfected SVGA astrocytes studied in vitro, produces IL-6 in astrocytes that further aggravates neuroinflammation (Yu et al., 2017; Shah et al., 2012). Another study by Mahajan et al. further supported the statement that HIV and meth increase the permeability of the BBB using in vitro cultures of BVECs. Cells treated with HIV gp120 and meth showed decreased levels of transendothelial electrical resistance (TEER), a measure of ion movement across the BBB (permeability), compared to cells treated with gp120 or meth alone (Mahajan et al., 2008a). By disrupting the BBB, meth and HIV acting together increase the neuroinflammatory state that eventually contributes to cognitive decline in this cohort (Chilunda et al., 2019). Furthermore, in more recent studies investigating pathogenic mechanisms of HIV infection in the brain exacerbated by meth, syncytia formation and subsequent HIV infection was observed through the increasing of intercellular adhesion molecule-1 (ICAM-1) and HIV-Nef protein on extracellular vesicles released from both uninfected and chronically HIV-1 infected promonocytic U1 cells in response to meth (Chand et al., 2022). As described above, it is reasonable to conclude that physiological barriers may be altered resulting in dysfunction and increased infectivity through the actions of methamphetamines in the HIV-1 infected brain; whether it be through neuroinflammation, BBB permeability, or alteration in adhesion molecules.

Studying the involvement in the hippocampus, a site of spatial learning and long-term memory formation, Zheng et al., observed augmented LTP by gp120 secondary to meth, implicating a synergistic effect. Although LTP is an experimental analogue for learning used in vitro and electrophysiology studies, it suggests that this may be a contributing factor to neurocognitive decline observed in HAND (Zheng et al., 2022). Infection of HIV in neural progenitor cells (NPC), mainly localized in the hippocampal dentate gyrus and around the lateral walls of the lateral ventricles in an adult mouse brain (Bordiuk et al., 2014), can also impair neurogenesis and lead to acceleration of HAND (Skowronska et al., 2018). Skowronska and colleagues indicated that pretreatment, but not simultaneous exposure with meth on mouse and human NPCs increases viral replication (Skowronska et al., 2018). Treatment with meth also increases viral load in a rhesus macaque model (Marcondes et al., 2010) and specifically by ∼6 fold in an HIV transgenic mouse model (Toussi et al., 2009) when compared to controls. Further findings using an EcoHIV mouse model exposed to meth demonstrated that combined exposure exerts a long-term effect on neurogenesis in adult mice by enhancing NPCs proliferation, which can impair and induce an aberrant differentiation into neuronal and astrocytic lineages (Park et al., 2021; Skowronska et al., 2018; Krathwohl and Kaiser, 2004). These results may reflect because of increased glutamate levels and upregulation of neuroinflammation through infection of HIV and meth exposure in glial cells. Taken together, although the findings discussed in this section are results of different methods used to mimic the HIV-infected brain exposed to meth, the data suggest that the dysregulation of the functional capacities of astrocytes, via inducing inflammation, mitochondrial dysfunction, and expression modulation results in neuronal insult and neuropathogenesis, which may further aggravate addiction and HAND development.

### 4.2 Cocaine

Cocaine is another psychostimulant that is used by nearly 5.2 million people annually, ages 12 and older, in the US (Abuse, 2022). HIV infected individuals with a history or who currently use cocaine have been found to have a worse cognitive performance than people living with HIV that do not use cocaine (Meade et al., 2015). The same study showed that HIV positive patients who use cocaine tend to have a lower ART adherence. Moreover, studies with controlled ART adherence have shown that use of cocaine increases total viral load and display more robust viral rebound after ART discontinuation (Carrico, 2011; Carrico et al., 2007, 2008; Baum et al., 2009; Cook et al., 2008; Moore et al., 2004). Studies also using chimeric mouse (Roth et al., 2002, 2005) and BLT humanized mouse models (Kim et al., 2015) demonstrated an increased percentage of lymphocytes infected and increased viral load after daily cocaine administration and an increased expression of inflammatory cytokines, IFN-y and IL-6, after continuous cocaine administration, respectively. These studies prove that the use of cocaine has direct effects on the HIV cohort, demonstrating that poor adherence to HIV medication is not the main factor for disease progression.

Like other psychostimulants, cocaine has implications on astrocyte function and BBB integrity. In the context of HIV infection, the combined effects of HIV neurotoxins and cocaine are likely to be worse, indicating a need for further study. HIV and cocaine disrupt the downstream pathway of LXR signaling and effectively reduce expression of the protein products of this pathway such as ApoE, ABCa1, and HMG-CoA reductase, proteins that are involved in the metabolism of cholesterol (Cotto et al., 2018). Neurons cultured with astrocytes that were treated with Tat and cocaine demonstrated increased levels of low-density lipoprotein receptors (LDLR), which suggests that there are in fact cholesterol deficits in the CNS in presence of HIV and cocaine. Decreased cholesterol availability along with decreased levels of synaptic proteins observed in the study module leads to disrupted synaptic connectivity (Cotto et al., 2018). These findings strongly suggest that HIV and cocaine work in a synergistic manner to disrupt cholesterol metabolism in the CNS, resulting in neurodegeneration and cognitive impairments. Meanwhile, studies have shown that cocaine and Tat both lead to overexcitation of pyramidal neurons in rat models; additionally they have demonstrated that Tat-induced neuronal excitation occurs in lower doses of Tat in rats that self-administered cocaine (Wayman et al., 2015). This model shows another possible way through which cocaine and HIV proteins lead to worse cognitive outcomes in patients with HIV that concomitantly use cocaine.

Independently, cocaine and HIV impair astroglial functions or morphology and are shown to increase glial fibrillary acidic protein (GFAP) expression, and in an additive manner when combined (Yang et al., 2016). Postmortem analysis of HIV positive patients with a history of cocaine use exhibited a significant increase in GFAP positive cells in cortical brain sections compared to HIV positive individuals that did not consume cocaine (Yang et al., 2016). A preclinical study showed that dentate gyrus tissue of mice 24 h after a single cocaine injection had strong expression of GFAP and decreased astrocytic size, these changes were observed in both human and rodent astrocytic cells (Fattore et al., 2002). A study done using astrocytic cell lines found that cocaine can cause endoplasmic reticulum (ER) stress induction of autophagy that correlated with GFAP upregulation and increased expression of TNF, IL1B, and IL6 (Periyasamy et al., 2016). Yang and colleagues found another mechanism involving the translocation of sigma-1 receptor to the plasma membrane of mouse primary astrocytes treated with cocaine in culture that further caused downstream activation of early growth response gene 1 (Egr-1) and subsequent activation of GFAP. These findings were further validated using male mice injected with cocaine that resulted in increased GFAP and Egr-1 expression in brain cortex compared to saline injected mice (Yang et al., 2016). Enhanced GFAP expression is an example of reactive astrocytosis, a hallmark of HAND progression (Sacktor et al., 2001). Astrocyte activation may not only lead to the release of cytokines and chemokines, but also other neurotoxic factors such as reactive oxidative stress (ROS) that are dangerous to neurons (Yang et al., 2016). Cocaine and HIV proteins, including gp120 and Tat, are reported to affect brain homeostasis by altering astrocyte metabolic function by increased ROS production (Natarajaseenivasan et al., 2018) and reducing lactate shuttling to neurons (Yang et al., 2010; Xu et al., 2012), inducing neuronal damage and even apoptosis. Cocaine and HIV proteins have shared pathways that lead to a chronic inflammatory state that negatively impacts the CNS.

As already mentioned in sections above, viral protein exposure of HIV has implications in the CNS, dysregulating the dopaminergic and glutamatergic state, and directly causes neuronal injury, altering cognitive function. However, the use of single HIV viral proteins to properly study the comorbidity of cocaine use in HIV-1 is a limitation. One study using a female and male EcoHIV mouse model evaluated the anatomical structure of pyramidal neurons in the medial PFC after a self-administration paradigm. The authors found that EcoHIV mice that self-administered cocaine or sucrose had a disruption in their decision making between the two rewards and blunted extinction learning. This data is consistent with another study using an HIV-1 transgenic rat model, where the presence of HIV-1 disrupted the choice preference between cocaine and sucrose in a self-administration paradigm (Bertrand et al., 2018). Furthermore, McLaurin and colleagues found that the EcoHIV model had an increased frequency of dendritic spines along the apical dendrite, with the spines having an increased head and neck diameter in the mPFC, compared to saline rats (McLaurin et al., 2022). Collectively, these data suggest that infection with HIV-1 can alter synaptic connectivity in the frontal-striatal circuit and increase the formation of immature dendritic spines in the mPFC. This structural reorganization of frontal-striatal circuit can disrupt glial-synapse interactions. Having stated this, more studies of these shared pathways should be performed in order to appropriately manage patients with HAND and concomitant substance abuse disorder in hopes of reducing the morbidity in said patients.

### 4.3 Opioids

Opioid use in the US has reached national crisis levels and overdose deaths (49,860 deaths annually according to the NIH) exceed those reported for methamphetamines or cocaine (Mattson et al., 2021; SAMHSA, 2020). According to the National Institute on Drug abuse, more than 90 Americans die by opioid overdose every day (Anesthesiologists, 2022). Although opiates are very efficacious analgesics for medical use, they are increasingly misused for recreational purposes (Corkrum et al., 2019). Over half of PLWH experience chronic pain in their lifetimes, particularly peripheral neuropathy that is undertreated and complex to manage (Madden et al., 2020; Addis et al., 2020; Krashin et al., 2012). Heroin, a semisynthetic opioid used recreationally, is metabolized into its main bioactive product, morphine, the analgesic most frequently prescribed by physicians for treatment of severe pain in HIV individuals. Opiate use in the context of HIV infection has been seen to worsen the existing chronic CNS inflammation and increase the speed of disease progression (Festa and Meucci, 2012; Barbour et al., 2020). Although cross-sectional observational studies dispute the fact that opioids worsen neuroHIV or HAND, there are significant cumulative in vitro and in vivo studies referenced below that suggest otherwise (Martin et al., 2019; Fitting et al., 2020). For example, Cornwell et al. used NSG humanized mice to study the immunomodulatory effects of tobacco and morphine in the brain during HIV infection. In the context of morphine specifically, they found that combined exposure reduced CD4 and increased CD8 counts by 8 weeks in HIV- infected mice compared to HIV alone, which prove disease progression (Cornwell et al., 2020). In a study using an SIV macaque model of HIV, those also treated with morphine had increased migration of monocytes into the brain compared to SIV monkeys alone (Bokhari et al., 2011), suggesting that HIV and morphine induce cell activation and inflammation, increasing monocyte entry into the CNS. For this reason, in the scientific studies we highlight in this section, opioids and their effects on the HIV infected brain use morphine as a proxy to demonstrate opioid effects.

The incidence of neuroHIV and CNS complications has increased among people living with HIV (PLWH) with opioid use disorder (OUD) (Smith et al., 2014). Chronic opioid use alone, was found to cause leukoencephalopathy, atrophy, and an increase in hyperphosphorylated tau neurofibrillary tangles compared to age-matched controls (Ramage et al., 2005; Cadet et al., 2014). Opioid and chemokine receptors are constitutively expressed in the CNS, and many studies suggest there may be crosstalk, leading to synergism or additive effects, between HIV and opioids (Festa and Meucci, 2012).

Opioids activate mu, kappa, and delta receptors, which are inhibitory G-protein coupled receptors (Gi/o) that carry out different functions depending on where they are found (Al-Hasani and Bruchas, 2011). Astrocytes in the hippocampus, VTA, and NAc express these opioid receptors (Nam et al., 2018). Although demonstrated through various methods of experimental design, opiates have been shown to lead to neurotoxicity through distinct mechanisms, such as ion transporter and neurotransmitter dysfunction, synergistic activity with HIV proteins mediated through cell surface receptors, and through dysregulation of protein levels (Kim et al., 2018a; Festa and Meucci, 2012; Barbour et al., 2020). Opioid effects in long term users have shown to cause astrogliosis, a natural response to CNS injury causing functional alterations in reactive astrocytes in attempt to minimize damage and promote repair (Sofroniew, 2014; Spencer et al., 2022). They also increase proinflammatory cytokines and inflammatory mediators such as TNF-α, IL-1β, and nitric oxide synthase (Dyuizen and Lamash, 2009). A key point of convergence between HIV and opiate use is the oxidative damage in the brain. Additionally, CD4 counts have been found to decrease further in response to opioid use in HIV positive individuals who are not adherent to ART (Meijerink et al., 2014). Even in the presence of ART, opioids have contributed to neuropathology, CNS inflammation, and deficits in cognition (Fitting et al., 2020). Studies have shown an increase in expression of GFAP in the VTA in response to opioids, suggesting a direct relation between astrocytes and opioids. Prior evidence demonstrated associations between opioids and a decreased expression of the astrocyte glutamate transporter GLT1 in both the thalamus and striatum. Observation that GLT1 activation decreases drug related behaviors as seen in Nakagawa et al., 2005, has shed light on an important potential piece of the puzzle to the drug use problems seen in opioid dependency by directly linking astrocytes and drug addiction behavior through mouse modeling and immunohistochemistry (Corkrum et al., 2019; Nakagawa et al., 2005). According to a study conducted by Hahn et al., 2016), induction of central HIV-1 Tat in mice correlated with behavioral modifications observed in amygdala function such as fear conditioning and anxiety (open field and elevated plus maze), in the absence of any changes in baseline motor behaviors. Furthermore, in the same study, Tat expression was found to decrease maximal G-protein stimulation by MOR agonists in membranes from the forebrain, suggesting that if applied to human subjects, HIV positive drug users may take more of the drugs to produce similar euphoric effects (Hahn et al., 2016). Therefore, in summation of the aforementioned findings, it is important to recognize the role opioids play in creating neurotoxicity and neuroinflammation, their potential to decrease CD4 counts, and their influence on the increased observed drug seeking behaviors in this population.

Similar to meth, opiates induce changes in the BBB that lead to increased permeability and barrier dysfunction. These changes promote increased neuroinflammation and cognitive impairments. Mahajan and colleagues, studying the effects of morphine and HIV-1 Tat protein on BBB integrity, found that human brain microvascular endothelial cells (BMVECs) treated with coexposure of morphine and Tat showed a marked decrease in TEER. Additionally, when exposed to both, studies showed significantly lower levels of zonula occludens-1 protein (ZO-1) and occludin expression compared to control. Taken together, these results suggest that the opioids use in the context of HIV infection disrupts the integrity and function of the BBB by downregulating the expression of junctional proteins (Mahajan et al., 2008b). This proposed pathological mechanism may facilitate the transmigration and establishment of infected monocytes in the central nervous system, therefore enhancing the pathogenesis of neuro-HIV (Cotto et al., 2018).

Moreover, opioid use has been found to have significant adverse effects on treatment in HIV and shown to lead to non-adherence and decreased antiretroviral efficacy. Interestingly, morphine has been found to counteract effects of antiretroviral drugs. In a study conducted by Rodriguez et al., 2019), co-exposure of opioids with ARVs in HIV infected astrocytes was shown to induced mitochondrial compromise and epigenetic changes, that may explain elevated viral titer and inflammation observed in HIV neuropathologies (Rodriguez et al., 2019). Supported findings from a previous study conducted by Vaidya et al., investigating ARVs efficacy in SIV-infected macaques as a proxy for HIV in humans, suggest that these observed effects may be due to an increased expression of HIV co-receptors CCR5 and CXCR4 in the periphery. This study revealed ART failed to control the viral loads in morphine-dependent animals (Vaidya et al., 2016). Furthermore, investigating this on an astrocyte driven level, the authors reported that in the presence of morphine in conjunction with protease inhibitors, reversal of the previous established reductions in HIV infected astrocytes were observed.

In summation, whether taken in the setting of prescribed or non-prescribed pain control or as a recreation euphoric drug, opioid use has significant and widespread effects on the HIV infected brain. In addition to mediating its proposed effects through oxidative damage, pro-inflammatory inducing states resulting in astrogliosis and physiological changes such as disruption of the BBB integrity, it has been implicated as in behavioral modification and drug seeking. The implications of the interconnecting effects between opioid use and HIV infection in the brain, although commonly supported as mentioned above through experimental models that serve as analogues to what may be happening within the human brain, should raise awareness and spark discussion to work on addressing the health care prescription and support provided to the HIV positive individuals who are chronic users and at high risk of developing neurocognitive deficits.

## 5 Discussion

The synergistic events that interweave the HIV and substance use epidemics worsen patient health status. Since HIVs discovery in the 1980s, advances in the study of HIV have allowed PLWH near-normal life expectancies; however, the cohort of HIV-positive patients that use addictive substances have more severe outcomes than those who do not use drugs. Patients with substance use disorder experience faster cognitive decline with associated severe neuroinflammation and progression of HAND (Tyagi et al., 2016; Yu et al., 2017; Festa and Meucci, 2012). The overlapping alterations of CNS homeostasis and neural plasticity by HIV and drug use are increasingly detrimental to astrocytes. Granting the wide debate over astrocyte role as HIV reservoir due to insufficient CD4 expression and the inefficient in vitro infection (Woodburn et al., 2022; Li et al., 2020; Edara et al., 2020), once the barrier of viral entry is overcome, stable provirus integration changes astrocyte phenotype and a productive infection is established based on viral promoter (Malik et al., 2021; Borrajo et al., 2021a; Edara et al., 2020). Clear in vivo transmission to astrocytes and their abundance in the CNS suggest that even low infection rates could have profound effects (Valdebenito et al., 2021; Lutgen et al., 2020). Understanding astrocytic dysfunction within this confluence of diverse interactions is thus a crucial step towards advancing therapeutic measures. We present an organized review regarding astrocyte function within neural circuitry and in response to the effects of HIV and substance use with the intention of summarizing current understanding, to delineate areas for future investigations, and to provide a path for novel preventative and therapeutic strategies afflicting this subpopulation of individuals living with HIV.

**Fig. 3:**
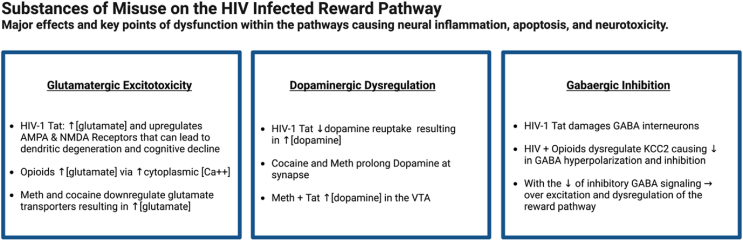
Overview of proposed effects of CNS Damage by HIV and Drugs: Methamphetamines, Cocaine, and Opioids.

Astrocyte impairment is particularly unfavorable for stimulus-induced brain plasticity, as it results in maladaptive neuronal responses and worsening of addiction behaviors (Scofield et al., 2015; Gomez et al., 2019; Koob and Volkow, 2016; Aksenov et al., 2012). Both viral proteins Tat and gp120 have been found to increase reward cues for select drugs that act with the viral components to alter neuronal density and transmission (Kesby et al., 2016, 2017; Wayman et al., 2016; McIntosh et al., 2015; Bansal et al., 2000). Substance use is both a prevalent comorbidity and an important risk factor in acquiring HIV (Nestler, 2005) that exacerbates BBB damage, cell transmigration and, pro-inflammatory peptide release even in the presence of ART. Therefore, in PLWH, there is increased susceptibility to the development of SUD which is incremented further upon starting therapeutic opioid regimens to manage pain. Viral proteins and drugs produce convergent damage to dopaminergic, glutamatergic, and GABAergic neurons of the reward pathway leading to synaptic dysregulation (Wang et al., 2004; Fitting et al., 2010b, 2013, 2014; Berridge, 1987; Marks et al., 2016; Barbour et al., 2020). Neuroinflammation exacerbates synaptic damage by abating astrocytic maintenance of neural and synaptic homeostasis. HIV patients with severe HAND are notably vulnerable to dysregulation due to decreased dopaminergic projections and transporters, leading to hyperexcitability and dendritic damage that worsen neurocognitive decline and SUD (Wang et al., 2004; Chang et al., 2008; Zhu et al., 2009; Wallace et al., 2006; Gelman et al., 2012). Experiments show Tat protein reinforces drug self-administration, as seen in HIV-1 transgenic rats later found to have modified dopamine transporter function, suggesting the groundwork for the overlapping CNS deterioration (McIntosh et al., 2015; Bertrand et al., 2018; Bansal et al., 2000). Elevated glutamate concentrations, which are produced by enduring neuroinflammation, upregulation of AMPARs and NMDARs (Haughey et al., 2001; Mattson et al., 2005) and viral Tat protein, lead to the dendritic shortening commonly found in HIV-1 patients. This shortening can contribute to synapto-dendritic degeneration upon co-exposure to opiates due to disruption of Ca2+ homeostasis (Fitting et al., 2010a, 2014). Glutamate overflow into the NAc is proposed to magnify reinstatement of drug-seeking (Buch et al., 2012) most likely secondary to astrocyte mediated glutamate transporter downregulation found in primary human astrocytes exposed to HIV and methamphetamine (Cisneros and Ghorpade, 2012). Furthermore, researchers correlated stressor-related glutamate excitotoxicity, in addition to the established role of elevations by HIV, with cognitive decline in patients with HAND (Cassol et al., 2014; Ferrarese et al., 2001). CNS hyperexcitation is further complicated by the targeted destruction of GABAergic interneuron gene markers and electrolyte transporters, as seen in the HIV Tat transgenic mouse model brain tissue and in autopsy samples from HIV-infected patients (Fitting et al., 2013; Marks et al., 2016; Barbour et al., 2020). This suggests that the HIV-infected brain has a propensity for synaptic dysregulation that supports substance use which intrinsically sustains the neuroinflammatory changes associated with HAND progression and addiction reinforcement. Highlighted in Fig. 3, we present an overview of the proposed mechanisms by which select drugs exert their effects in the HIV infected brain.

Substance use also poses a complex clinical challenge: ART adherence vs. opioid therapy for pain management. Although general use of opioids has decreased, morphine is commonly prescribed for PLWH that experience severe pain despite opiate interference with antiretroviral drug adherence (Hahn et al., 2016) and hastened CD4 count decline in ART non-adherent patients (Meijerink et al., 2014). Additionally, the synergistic effects of HIV and substance use are such that, even when ART is taken properly, opioids cause inflammation and degeneration in the CNS though some debate this finding (Martin et al., 2019). Of note, multiple studies suggest that opioid-exposed astrocytes counteract anti-viral medications leading to poorly controlled viral loads (Rodriguez et al., 2019; Fitting et al., 2020; Vaidya et al., 2016). Opioids, as well as the stimulants cocaine and meth, precipitate reactive astrogliosis (reflected by overexpression of GFAP) (Sofroniew, 2014; Spencer et al., 2022); a regular finding in progressive HAND (Sacktor et al., 2001; Yang et al., 2010). In the HIV-infected brain, these drugs further disrupt homeostasis by depleting CNS cholesterol (Cotto et al., 2018), synaptic proteins (Fattore et al., 2002), ion transporters, surface receptors (Barbour, 2020; Fattore, 2002; Festa and Meucci, 2012; Kim et al., 2018b) and by inducing astrocyte death via mitochondrial dysfunction and oxidative stress (Borgmann and Ghorpade, 2018). HIV and drugs like opioids and meth have been to synergistically disrupt the BBB (Mahajan et al., 2008a) facilitating infected monocyte transmigration (Cotto et al., 2018) and inducing a neuroinflammation process that leads to cognitive decline (Chilunda et al., 2019). With the availability of human studies and improved model systems just now growing, the limitation of experimentation in the monoculture setting can be addressed to establish a clearer connection between HIV, SUD and astrocyte function within the CNS resident cell ecosystem. For this reason, we encourage future researchers to investigate the role of astrocytes within these synergistic pathways for the development of complementary treatments to ART for the management of neuroinflammation, HAND progression, and substance use cessation.

## 6 Conclusion

The extensive effect of viral proteins on neurocircuitry and the propensity for establishment/enhancement of addiction is fundamental when considering patient risk in PLWH. Endurance of stable circuitry reduces glial exacerbation of addictive behavior, the additive deleterious effect of substance use and the progression of HAND. Consideration of addiction extinction via maintenance of astrocyte homeostasis may serve to improve HIV prognosis and adherence to therapeutic regimen for more effective healthcare.

## Ethics statement

The work presented did not involve human or animal subjects. Declarations of interest: none. This work has not been published elsewhere in any format and is not under consideration for publication elsewhere. All authors have approved of the current version of the manuscript for submission.

## Funding

Support for this work was provided by the RCMI Specialized Center for Health Disparities at PHSU (MD007579) and the Ponce Research Institute (MD summer research program for LAS and PAGC). JPT was supported by GM082406 and F31DA054814. Figures were created with BioRender.com by AMR, publication licenses were obtained on January 26, 2023.

## CRediT authorship contribution statement

**Jessalyn Pla-Tenorio:** Conceptualization, Data curation, Formal analysis, Project administration, Writing – original draft, Writing – review & editing. **Angela M. Roig:** Conceptualization, Data curation, Formal analysis, Project administration, Visualization, Writing – original draft, Writing – review & editing. **Paulina A. García-Cesaní:** Conceptualization, Data curation, Formal analysis, Writing – original draft, Writing – review & editing. **Luis A. Santiago:** Conceptualization, Data curation, Formal analysis, Writing – original draft, Writing – review & editing. **Marian T. Sepulveda-Orengo:** Supervision, Writing – review & editing. **Richard J. Noel:** Funding acquisition, Supervision, Writing – original draft, Writing – review & editing.

## Declaration of competing interest

The authors declare that they have no known competing financial interests or personal relationships that could have appeared to influence the work reported in this paper.

## Data Availability

No data was used for the research described in the article.

## References

[bib1] Abuse N.I.o.D. (2022).

[bib2] Addis D.R., DeBerry J.J., Aggarwal S. (2020). Chronic pain in HIV. Mol. Pain.

[bib3] Aksenov M.Y. (2012). D1/NMDA receptors and concurrent methamphetamine+ HIV-1 Tat neurotoxicity. J. Neuroimmune Pharmacol..

[bib4] Al-Hasani R., Bruchas M.R. (2011). Molecular mechanisms of opioid receptor-dependent signaling and behavior. Anesthesiology.

[bib5] Amara S.G., Sonders M.S. (1998). Neurotransmitter transporters as molecular targets for addictive drugs. Drug Alcohol Depend..

[bib6] Anesthesiologists A.S.o. (2022). *Opioid abuse*. Made for this moment. https://www.asahq.org/madeforthismoment/pain-management/opioid-treatment/opioid-abuse/#:~:text=Statistics%20highlight%20the%20severity%20of,overdose%20every%20day%2C%20on%20average.

[bib7] Antinori A. (2007). Updated research nosology for HIV-associated neurocognitive disorders. Neurology.

[bib8] Ash M.K., Al-Harthi L., Schneider J.R. (2021). HIV in the brain: identifying viral reservoirs and addressing the challenges of an HIV cure. Vaccines.

[bib9] Bairwa D. (2016). Case control study: magnetic resonance spectroscopy of brain in HIV infected patients. BMC Neurol..

[bib10] Baker D.A. (2002). The origin and neuronal function of in vivo nonsynaptic glutamate. J. Neurosci..

[bib11] Baker D.A. (2003). Neuroadaptations in cystine-glutamate exchange underlie cocaine relapse. Nat. Neurosci..

[bib12] Bansal A.K. (2000). Neurotoxicity of HIV-1 proteins gp120 and Tat in the rat striatum. Brain Res..

[bib13] Barbour A.J. (2020). HIV and opiates dysregulate K(+)- Cl(-) cotransporter 2 (KCC2) to cause GABAergic dysfunction in primary human neurons and Tat-transgenic mice. Neurobiol. Dis..

[bib14] Baum M.K. (2009). Crack-cocaine use accelerates HIV disease progression in a cohort of HIV-positive drug users. J. Acquir. Immune Defic. Syndr..

[bib15] Berridge J. (1987). Infection control and the states. J. Am. Dent. Assoc..

[bib16] Bertrand S.J. (2015). HIV-1 Tat and cocaine mediated synaptopathy in cortical and midbrain neurons is prevented by the isoflavone Equol. Front. Microbiol..

[bib17] Bertrand S.J. (2018). HIV-1 proteins dysregulate motivational processes and dopamine circuitry. Sci. Rep..

[bib18] Bokhari S.M. (2011). Morphine potentiates neuropathogenesis of SIV infection in rhesus macaques. J. Neuroimmune Pharmacol..

[bib19] Bordiuk O.L. (2014). Cell proliferation and neurogenesis in adult mouse brain. PLoS One.

[bib20] Borgmann K., Ghorpade A. (2015). HIV-1, methamphetamine and astrocytes at neuroinflammatory Crossroads. Front. Microbiol..

[bib21] Borgmann K., Ghorpade A. (2018). Methamphetamine augments concurrent astrocyte mitochondrial stress, oxidative burden, and antioxidant capacity: tipping the balance in HIV-associated neurodegeneration. Neurotox. Res..

[bib22] Borrajo A. (2021). Crucial role of central nervous system as a viral anatomical compartment for HIV-1 infection. Microorganisms.

[bib23] Borrajo A. (2021). Important role of microglia in HIV-1 associated neurocognitive disorders and the molecular pathways implicated in its pathogenesis. Ann. Med..

[bib24] Bouwman F.H. (1998). Variable progression of HIV-associated dementia. Neurology.

[bib25] Bowers M.S. (2004). Activator of G protein signaling 3: a gatekeeper of cocaine sensitization and drug seeking. Neuron.

[bib26] Brodie J.D., Figueroa E., Dewey S.L. (2003). Treating cocaine addiction: from preclinical to clinical trial experience with gamma-vinyl GABA. Synapse.

[bib27] Buch S. (2012). Cocaine and HIV-1 interplay in CNS: cellular and molecular mechanisms. Curr. HIV Res..

[bib28] Buzhdygan T. (2016). Neuropsychological, neurovirological and neuroimmune aspects of abnormal GABAergic transmission in HIV infection. J. Neuroimmune Pharmacol..

[bib29] Cadet J.L., Bisagno V., Milroy C.M. (2014). Neuropathology of substance use disorders. Acta Neuropathol..

[bib30] Carrico A.W. (2011). Substance use and HIV disease progression in the HAART era: implications for the primary prevention of HIV. Life Sci..

[bib31] Carrico A.W. (2007). Affect regulation, stimulant use, and viral load among HIV-positive persons on anti-retroviral therapy. Psychosom. Med..

[bib32] Carrico A.W. (2008). Stimulant use is associated with immune activation and depleted tryptophan among HIV-positive persons on anti-retroviral therapy. Brain Behav. Immun..

[bib33] Cassol E. (2014). Cerebrospinal fluid metabolomics reveals altered waste clearance and accelerated aging in HIV patients with neurocognitive impairment. AIDS.

[bib34] Chand S. (2022). Methamphetamine induces the release of proadhesive extracellular vesicles and promotes syncytia formation: a potential role in HIV-1 neuropathogenesis. Viruses.

[bib35] Chang L. (2008). Decreased brain dopamine transporters are related to cognitive deficits in HIV patients with or without cocaine abuse. Neuroimage.

[bib36] Channer B. (2022).

[bib37] Chen J. (2020). Chemokine CCL2 impairs spatial memory and cognition in rats via influencing inflammation, glutamate metabolism and apoptosis-associated genes expression- a potential mechanism for HIV-associated neurocognitive disorder. Life Sci..

[bib38] Chen G. (2020). Methamphetamine inhibits long-term memory acquisition and synaptic plasticity by evoking endoplasmic reticulum stress. Front. Neurosci..

[bib39] Cheng J. (1998). Neuronal excitatory properties of human immunodeficiency virus type 1 Tat protein. Neuroscience.

[bib40] Chilunda V. (2019). The impact of substance abuse on HIV-mediated neuropathogenesis in the current ART era. Brain Res..

[bib41] Chompre G. (2013). Astrocytic expression of HIV-1 Nef impairs spatial and recognition memory. Neurobiol. Dis..

[bib42] Cisneros I.E., Ghorpade A. (2012). HIV-1, methamphetamine and astrocyte glutamate regulation: combined excitotoxic implications for neuro-AIDS. Curr. HIV Res..

[bib43] Cook J.A. (2008). Crack cocaine, disease progression, and mortality in a multicenter cohort of HIV-1 positive women. AIDS.

[bib44] Corkrum M. (2019). Opioid-mediated astrocyte-neuron signaling in the nucleus accumbens. Cells.

[bib45] Cornwell W.D. (2020). Tobacco smoke and morphine alter peripheral and CNS inflammation following HIV infection in a humanized mouse model. Sci. Rep..

[bib46] Cotto B. (2018). Cocaine and HIV-1 Tat disrupt cholesterol homeostasis in astrocytes: implications for HIV-associated neurocognitive disorders in cocaine user patients. Glia.

[bib47] Daberkow D.P. (2008). Effect of methamphetamine neurotoxicity on learning-induced Arc mRNA expression in identified striatal efferent neurons. Neurotox. Res..

[bib48] Dewey S.L. (1998). A novel strategy for the treatment of cocaine addiction. Synapse.

[bib49] Dewey S.L. (1999). A pharmacologic strategy for the treatment of nicotine addiction. Synapse.

[bib50] Doyon N. (2016). Chloride regulation: a dynamic equilibrium crucial for synaptic inhibition. Neuron.

[bib51] Dyuizen I., Lamash N.E. (2009). Histo- and immunocytochemical detection of inducible NOS and TNF-alpha in the locus coeruleus of human opiate addicts. J. Chem. Neuroanat..

[bib52] Edara V.V., Ghorpade A., Borgmann K. (2020). Insights into the gene expression profiles of active and restricted red/green-HIV(+) human astrocytes: implications for shock or lock therapies in the brain. J. Virol..

[bib53] Elgueta D. (2017). Pharmacologic antagonism of dopamine receptor D3 attenuates neurodegeneration and motor impairment in a mouse model of Parkinson's disease. Neuropharmacology.

[bib54] Ernst T. (2010). Lower brain glutamate is associated with cognitive deficits in HIV patients: a new mechanism for HIV-associated neurocognitive disorder. J. Magn. Reson. Imag..

[bib55] Farber K., Pannasch U., Kettenmann H. (2005). Dopamine and noradrenaline control distinct functions in rodent microglial cells. Mol. Cell. Neurosci..

[bib56] Fattore L. (2002). Astroglial in vivo response to cocaine in mouse dentate gyrus: a quantitative and qualitative analysis by confocal microscopy. Neuroscience.

[bib57] Ferrarese C. (2001). Increased glutamate in CSF and plasma of patients with HIV dementia. Neurology.

[bib58] Festa L., Meucci O. (2012). Effects of opiates and HIV proteins on neurons: the role of ferritin heavy chain and a potential for synergism. Curr. HIV Res..

[bib59] Fitting S. (2010). Interactive comorbidity between opioid drug abuse and HIV-1 Tat: chronic exposure augments spine loss and sublethal dendritic pathology in striatal neurons. Am. J. Pathol..

[bib60] Fitting S. (2010). Dose-dependent long-term effects of Tat in the rat hippocampal formation: a design-based stereological study. Hippocampus.

[bib61] Fitting S. (2013). Synaptic dysfunction in the hippocampus accompanies learning and memory deficits in human immunodeficiency virus type-1 Tat transgenic mice. Biol. Psychiatr..

[bib62] Fitting S. (2014). Interactive HIV-1 Tat and morphine-induced synaptodendritic injury is triggered through focal disruptions in Na(+) influx, mitochondrial instability, and Ca(2)(+) overload. J. Neurosci..

[bib63] Fitting S., McRae M., Hauser K.F. (2020). Opioid and neuroHIV comorbidity - current and future perspectives. J. Neuroimmune Pharmacol..

[bib64] Franz D. (2015). Dopamine receptors D3 and D5 regulate CD4(+)T-cell activation and differentiation by modulating ERK activation and cAMP production. J. Neuroimmunol..

[bib65] Galea I. (2021). The blood-brain barrier in systemic infection and inflammation. Cell. Mol. Immunol..

[bib66] Gelman B.B. (2012). Prefrontal dopaminergic and enkephalinergic synaptic accommodation in HIV-associated neurocognitive disorders and encephalitis. J. Neuroimmune Pharmacol..

[bib67] Gerasimov M.R. (1999). Gamma-vinyl GABA inhibits methamphetamine, heroin, or ethanol-induced increases in nucleus accumbens dopamine. Synapse.

[bib68] Giacometti L.L., Barker J.M. (2019). Comorbid HIV infection and alcohol use disorders: converging glutamatergic and dopaminergic mechanisms underlying neurocognitive dysfunction. Brain Res..

[bib69] Gomez J.A. (2019). Ventral tegmental area astrocytes orchestrate avoidance and approach behavior. Nat. Commun..

[bib70] Hahn Y.K. (2016). Central HIV-1 Tat exposure elevates anxiety and fear conditioned responses of male mice concurrent with altered mu-opioid receptor-mediated G-protein activation and β-arrestin 2 activity in the forebrain. Neurobiol. Dis..

[bib71] Han D.D., Gu H.H. (2006). Comparison of the monoamine transporters from human and mouse in their sensitivities to psychostimulant drugs. BMC Pharmacol..

[bib72] Hargus N.J., Thayer S.A. (2013). Human immunodeficiency virus-1 Tat protein increases the number of inhibitory synapses between hippocampal neurons in culture. J. Neurosci..

[bib73] Haughey N.J. (2001). HIV-1 Tat through phosphorylation of NMDA receptors potentiates glutamate excitotoxicity. J. Neurochem..

[bib74] Huck J.H. (2015). De novo expression of dopamine D2 receptors on microglia after stroke. J. Cerebr. Blood Flow Metabol..

[bib75] Illenberger J.M. (2020). HIV infection and neurocognitive disorders in the context of chronic drug abuse: evidence for divergent findings dependent upon prior drug history. J. Neuroimmune Pharmacol..

[bib76] Jaureguiberry-Bravo M., Lopez L., Berman J.W. (2018). Frontline Science: buprenorphine decreases CCL2-mediated migration of CD14(+) CD16(+) monocytes. J. Leukoc. Biol..

[bib77] Javadi-Paydar M. (2017). HIV-1 and cocaine disrupt dopamine reuptake and medium spiny neurons in female rat striatum. PLoS One.

[bib78] Jones S.R., Garris P.A., Wightman R.M. (1995). Different effects of cocaine and nomifensine on dopamine uptake in the caudate-putamen and nucleus accumbens. J. Pharmacol. Exp. Therapeut..

[bib79] Kang S. (2020). Activation of astrocytes in the dorsomedial striatum facilitates transition from habitual to goal-directed reward-seeking behavior. Biol. Psychiatr..

[bib80] Kesby J.P., Markou A., Semenova S. (2016). The effects of HIV-1 regulatory TAT protein expression on brain reward function, response to psychostimulants and delay-dependent memory in mice. Neuropharmacology.

[bib81] Kesby J.P. (2017). HIV-1 TAT protein enhances sensitization to methamphetamine by affecting dopaminergic function. Brain Behav. Immun..

[bib82] Kim H.J., Martemyanov K.A., Thayer S.A. (2008). Human immunodeficiency virus protein tat induces synapse loss via a reversible process that is distinct from cell death. J. Neurosci..

[bib83] Kim S.G. (2015). Cocaine-mediated impact on HIV infection in humanized BLT mice. Sci. Rep..

[bib84] Kim S. (2018). A central role for glial CCR5 in directing the neuropathological interactions of HIV-1 Tat and opiates. J. Neuroinflammation.

[bib85] Kim R. (2018). Regulation of glutamate transporter 1 (GLT-1) gene expression by cocaine self-administration and withdrawal. Neuropharmacology.

[bib86] Klawonn A.M., Malenka R.C. (2018). Nucleus accumbens modulation in reward and aversion. Cold Spring Harbor Symp. Quant. Biol..

[bib87] Koob G.F., Volkow N.D. (2016). Neurobiology of addiction: a neurocircuitry analysis. Lancet Psychiatr..

[bib88] Krashin D.L., Merrill J.O., Trescot A.M. (2012). Opioids in the management of HIV-related pain. Pain Physician.

[bib89] Krathwohl M.D., Kaiser J.L. (2004). HIV-1 promotes quiescence in human neural progenitor cells. J. Infect. Dis..

[bib90] Kumar U., Patel S.C. (2007). Immunohistochemical localization of dopamine receptor subtypes (D1R-D5R) in Alzheimer's disease brain. Brain Res..

[bib91] Kushner S.A., Dewey S.L., Kornetsky C. (1999). The irreversible gamma-aminobutyric acid (GABA) transaminase inhibitor gamma-vinyl-GABA blocks cocaine self-administration in rats. J. Pharmacol. Exp. Therapeut..

[bib92] Lemons A. (2019). Opioid misuse among HIV-positive adults in medical care: results from the medical monitoring project, 2009-2014. J. Acquir. Immune Defic. Syndr..

[bib93] Li Y. (1999). Both glutamate receptor antagonists and prefrontal cortex lesions prevent induction of cocaine sensitization and associated neuroadaptations. Synapse.

[bib94] Li G. (2010). Permeability of endothelial and astrocyte cocultures: in vitro blood-brain barrier models for drug delivery studies. Ann. Biomed. Eng..

[bib95] Li G.H. (2020). Productive HIV infection in astrocytes can be established via a nonclassical mechanism. AIDS.

[bib96] Luo X., He J.J. (2015). Cell-cell contact viral transfer contributes to HIV infection and persistence in astrocytes. J. Neurovirol..

[bib97] Lutgen V. (2020). HIV infects astrocytes in vivo and egresses from the brain to the periphery. PLoS Pathog..

[bib98] Madden V.J., Parker R., Goodin B.R. (2020). Chronic pain in people with HIV: a common comorbidity and threat to quality of life. Pain Manag..

[bib99] Mahajan S.D. (2008). Methamphetamine alters blood brain barrier permeability via the modulation of tight junction expression: implication for HIV-1 neuropathogenesis in the context of drug abuse. Brain Res..

[bib100] Mahajan S.D. (2008). Tight junction regulation by morphine and HIV-1 tat modulates blood-brain barrier permeability. J. Clin. Immunol..

[bib101] Malik S. (2021). HIV infection of astrocytes compromises inter-organelle interactions and inositol phosphate metabolism: a potential mechanism of bystander damage and viral reservoir survival. Prog. Neurobiol..

[bib102] Marcondes M.C. (2010). Methamphetamine increases brain viral load and activates natural killer cells in simian immunodeficiency virus-infected monkeys. Am. J. Pathol..

[bib103] Marks W.D. (2016). HIV-1 Tat causes cognitive deficits and selective loss of parvalbumin, somatostatin, and neuronal nitric oxide synthase expressing hippocampal CA1 interneuron subpopulations. J. Neurovirol..

[bib104] Martin E.M. (2019). Double dissociation of HIV and substance use disorder effects on neurocognitive tasks dependent on striatal integrity. AIDS.

[bib105] Mastroeni D. (2009). Microglial responses to dopamine in a cell culture model of Parkinson's disease. Neurobiol. Aging.

[bib106] Matejuk A., Ransohoff R.M. (2020). Crosstalk between astrocytes and microglia: an overview. Front. Immunol..

[bib107] Mathiisen T.M. (2010). The perivascular astroglial sheath provides a complete covering of the brain microvessels: an electron microscopic 3D reconstruction. Glia.

[bib108] Matt S.M. (2021). Dopamine levels induced by substance abuse alter efficacy of Maraviroc and expression of CCR5 conformations on myeloid cells: implications for NeuroHIV. Front. Immunol..

[bib109] Mattson M.P., Haughey N.J., Nath A. (2005). Cell death in HIV dementia. Cell Death Differ..

[bib110] Mattson C.L. (2021). Trends and geographic patterns in drug and synthetic opioid overdose deaths - United States, 2013-2019. MMWR Morb. Mortal. Wkly. Rep..

[bib111] McFarland K., Kalivas P.W. (2001). The circuitry mediating cocaine-induced reinstatement of drug-seeking behavior. J. Neurosci..

[bib112] McFarland K., Lapish C.C., Kalivas P.W. (2003). Prefrontal glutamate release into the core of the nucleus accumbens mediates cocaine-induced reinstatement of drug-seeking behavior. J. Neurosci..

[bib113] McIntosh S. (2015). Increased sensitivity to cocaine self-administration in HIV-1 transgenic rats is associated with changes in striatal dopamine transporter binding. J. Neuroimmune Pharmacol..

[bib114] McIntosh E.C. (2022). Prefrontal cortex volume mediates the relationship between lifetime chronic stressor exposure and cognition in people living with and without HIV. Psychosom. Med..

[bib115] McLaughlin J.P. (2014). HIV-1 Tat protein exposure potentiates ethanol reward and reinstates extinguished ethanol-conditioned place preference. Curr. HIV Res..

[bib116] McLaurin K.A. (2022). Disrupted decision-making: EcoHIV inoculation in cocaine dependent rats. Int. J. Mol. Sci..

[bib117] Meade C.S. (2015). Independent effects of HIV infection and cocaine dependence on neurocognitive impairment in a community sample living in the southern United States. Drug Alcohol Depend..

[bib118] Meijerink H. (2014). The number of CCR5 expressing CD4+ T lymphocytes is lower in HIV-infected long-term non-progressors with viral control compared to normal progressors: a cross-sectional study. BMC Infect. Dis..

[bib119] Miyazaki I. (2004). Direct evidence for expression of dopamine receptors in astrocytes from basal ganglia. Brain Res..

[bib120] Moore R.D., Keruly J.C., Chaisson R.E. (2004). Differences in HIV disease progression by injecting drug use in HIV-infected persons in care. J. Acquir. Immune Defic. Syndr..

[bib121] Musante V. (2010). The HIV-1 viral protein Tat increases glutamate and decreases GABA exocytosis from human and mouse neocortical nerve endings. Cerebr. Cortex.

[bib122] Najera J.A. (2016). Methamphetamine abuse affects gene expression in brain-derived microglia of SIV-infected macaques to enhance inflammation and promote virus targets. BMC Immunol..

[bib123] Nakagawa T. (2005). Effect of MS-153, a glutamate transporter activator, on the conditioned rewarding effects of morphine, methamphetamine and cocaine in mice. Behav. Brain Res..

[bib124] Nam M.H. (2018). Expression of micro-opioid receptor in CA1 hippocampal astrocytes. Exp Neurobiol.

[bib125] Narita M. (2006). Direct evidence of astrocytic modulation in the development of rewarding effects induced by drugs of abuse. Neuropsychopharmacology.

[bib126] Natarajaseenivasan K. (2018). Astrocytic metabolic switch is a novel etiology for Cocaine and HIV-1 Tat-mediated neurotoxicity. Cell Death Dis..

[bib127] Nath A. (2008). Evolution of HIV dementia with HIV infection. Int. Rev. Psychiatr..

[bib128] Nestler E.J. (2005). Is there a common molecular pathway for addiction?. Nat. Neurosci..

[bib129] Nickoloff-Bybel E.A. (2020). HIV neuropathogenesis in the presence of a disrupted dopamine system. J. Neuroimmune Pharmacol..

[bib130] Ota Y., Zanetti A.T., Hallock R.M. (2013). The role of astrocytes in the regulation of synaptic plasticity and memory formation. Neural Plast..

[bib131] Pacheco R., Contreras F., Zouali M. (2014). The dopaminergic system in autoimmune diseases. Front. Immunol..

[bib132] Paris J.J. (2014). Effects of conditional central expression of HIV-1 tat protein to potentiate cocaine-mediated psychostimulation and reward among male mice. Neuropsychopharmacology.

[bib133] Pariyadath V., Gowin J.L., Stein E.A. (2016). Resting state functional connectivity analysis for addiction medicine: from individual loci to complex networks. Prog. Brain Res..

[bib134] Park M. (2021). Methamphetamine enhances HIV-induced aberrant proliferation of neural progenitor cells via the FOXO3-mediated mechanism. Mol. Neurobiol..

[bib135] Pastuzyn E.D. (2012). Altered learning and Arc-regulated consolidation of learning in striatum by methamphetamine-induced neurotoxicity. Neuropsychopharmacology.

[bib136] Periyasamy P., Guo M.L., Buch S. (2016). Cocaine induces astrocytosis through ER stress-mediated activation of autophagy. Autophagy.

[bib137] Pierce R.C. (1998). Ibotenic acid lesions of the dorsal prefrontal cortex disrupt the expression of behavioral sensitization to cocaine. Neuroscience.

[bib138] Pinoli M., Marino F., Cosentino M. (2017). Dopaminergic regulation of innate immunity: a review. J. Neuroimmune Pharmacol..

[bib139] Plankey M.W. (2007). The relationship between methamphetamine and popper use and risk of HIV seroconversion in the multicenter AIDS cohort study. J. Acquir. Immune Defic. Syndr..

[bib140] Potter M.C. (2013). Targeting the glutamatergic system for the treatment of HIV-associated neurocognitive disorders. J. Neuroimmune Pharmacol..

[bib141] Prevention, C.f.D.C.a. (2021).

[bib142] Purohit V., Rapaka R., Shurtleff D. (2011). Drugs of abuse, dopamine, and HIV-associated neurocognitive disorders/HIV-associated dementia. Mol. Neurobiol..

[bib143] Purohit V. (2013). National Institute on Drug Abuse symposium report: drugs of abuse, dopamine, and HIV-associated neurocognitive disorders/HIV-associated dementia. J. Neurovirol..

[bib144] Qin Y. (2018). A milieu molecule for TGF-beta required for microglia function in the nervous system. Cell.

[bib145] Rahman M.M. (2022). Emerging role of neuron-glia in neurological disorders: at a glance. Oxid. Med. Cell. Longev..

[bib146] Ramage S.N. (2005). Hyperphosphorylated tau and amyloid precursor protein deposition is increased in the brains of young drug abusers. Neuropathol. Appl. Neurobiol..

[bib147] Ritz M.C. (1987). Cocaine receptors on dopamine transporters are related to self-administration of cocaine. Science.

[bib148] Rivera J. (2019). Infusion of HIV-1 Nef-expressing astrocytes into the rat hippocampus induces enteropathy and interstitial pneumonitis and increases blood-brain-barrier permeability. PLoS One.

[bib149] Rodriguez M. (2019). Morphine counteracts the antiviral effect of antiretroviral drugs and causes upregulation of p62/SQSTM1 and histone-modifying enzymes in HIV-infected astrocytes. J. Neurovirol..

[bib150] Roscoe R.F., Mactutus C.F., Booze R.M. (2014). HIV-1 transgenic female rat: synaptodendritic alterations of medium spiny neurons in the nucleus accumbens. J. Neuroimmune Pharmacol..

[bib151] Roth M.D. (2002). Cocaine enhances human immunodeficiency virus replication in a model of severe combined immunodeficient mice implanted with human peripheral blood leukocytes. J. Infect. Dis..

[bib152] Roth M.D. (2005). Cocaine and sigma-1 receptors modulate HIV infection, chemokine receptors, and the HPA axis in the huPBL-SCID model. J. Leukoc. Biol..

[bib153] Russell R.A. (2017). Astrocytes resist HIV-1 fusion but engulf infected macrophage material. Cell Rep..

[bib154] Sacktor N. (2001). HIV-associated neurologic disease incidence changes:: multicenter AIDS Cohort Study, 1990-1998. Neurology.

[bib155] Sailasuta N., Shriner K., Ross B. (2009). Evidence of reduced glutamate in the frontal lobe of HIV-seropositive patients. NMR Biomed..

[bib156] SAMHSA (2020).

[bib157] Sami Saribas A. (2017). HIV-1 Nef is released in extracellular vesicles derived from astrocytes: evidence for Nef-mediated neurotoxicity. Cell Death Dis..

[bib158] Saylor D. (2016). HIV-associated neurocognitive disorder - pathogenesis and prospects for treatment. Nat. Rev. Neurol..

[bib159] Schier C.J. (2017). Selective vulnerability of striatal D2 versus D1 dopamine receptor-expressing medium spiny neurons in HIV-1 tat transgenic male mice. J. Neurosci..

[bib160] Schifitto G. (2007). Memantine and HIV-associated cognitive impairment: a neuropsychological and proton magnetic resonance spectroscopy study. AIDS.

[bib161] Schmidt H.D., Famous K.R., Pierce R.C. (2009). The limbic circuitry underlying cocaine seeking encompasses the PPTg/LDT. Eur. J. Neurosci..

[bib162] Scofield M.D. (2015). Gq-DREADD selectively initiates glial glutamate release and inhibits cue-induced cocaine seeking. Biol. Psychiatr..

[bib163] Scofield M.D. (2016). The nucleus accumbens: mechanisms of addiction across drug classes reflect the importance of glutamate homeostasis. Pharmacol. Rev..

[bib164] Shah A. (2012). Synergistic cooperation between methamphetamine and HIV-1 gsp120 through the P13K/Akt pathway induces IL-6 but not IL-8 expression in astrocytes. PLoS One.

[bib165] Shao W. (2013). Suppression of neuroinflammation by astrocytic dopamine D2 receptors via alphaB-crystallin. Nature.

[bib166] Shin A.H., Thayer S.A. (2013). Human immunodeficiency virus-1 protein Tat induces excitotoxic loss of presynaptic terminals in hippocampal cultures. Mol. Cell. Neurosci..

[bib167] Silverman R.B. (2018). Design and mechanism of GABA aminotransferase inactivators. Treatments for epilepsies and addictions. Chem. Rev..

[bib168] Skowronska M. (2018). Methamphetamine increases HIV infectivity in neural progenitor cells. J. Biol. Chem..

[bib169] Smith C.J. (2014). Trends in underlying causes of death in people with HIV from 1999 to 2011 (D:A:D): a multicohort collaboration. Lancet.

[bib170] Sofroniew M.V. (2014). Astrogliosis. Cold Spring Harb Perspect Biol.

[bib171] Sofroniew M.V., Vinters H.V. (2010). Astrocytes: biology and pathology. Acta Neuropathol..

[bib172] Song L. (2003). Human immunodeficiency virus type 1 Tat protein directly activates neuronal N-methyl-D-aspartate receptors at an allosteric zinc-sensitive site. J. Neurovirol..

[bib173] Spencer A.C. (2022). Restoring the neuroprotective capacity of glial cells under opioid addiction. Addiction Neuroscience.

[bib174] Spurgat M.S., Tang S.J. (2022). Single-cell RNA-sequencing: astrocyte and microglial heterogeneity in health and disease. Cells.

[bib175] Stankoff B. (2001). Clinical and spectroscopic improvement in HIV-associated cognitive impairment. Neurology.

[bib176] Ton H., Xiong H. (2013). Astrocyte dysfunctions and HIV-1 neurotoxicity. J. AIDS Clin. Res..

[bib177] Toussi S.S. (2009). Short communication: methamphetamine treatment increases in vitro and in vivo HIV replication. AIDS Res. Hum. Retrovir..

[bib178] Tseng K.-Y., O'Donnell P. (2007). Dopamine modulation of prefrontal cortical interneurons changes during adolescence. Cerebr. Cortex.

[bib179] Tyagi M., Bukrinsky M., Simon G.L. (2016). Mechanisms of HIV transcriptional regulation by drugs of abuse. Curr. HIV Res..

[bib180] Vaidya N.K. (2016). Modeling the effects of morphine on simian immunodeficiency virus dynamics. PLoS Comput. Biol..

[bib181] Valdebenito S. (2021). Astrocytes are HIV reservoirs in the brain: a cell type with poor HIV infectivity and replication but efficient cell-to-cell viral transfer. J. Neurochem..

[bib182] Vazquez-Santiago F.J. (2014). Glutamate metabolism and HIV-associated neurocognitive disorders. J. Neurovirol..

[bib183] Ventuneac A. (2021). Chronic high risk prescription opioid use among persons with HIV. Front Sociol.

[bib184] Vera J.H. (2016). Neuroinflammation in treated HIV-positive individuals: a TSPO PET study. Neurology.

[bib185] Vivithanaporn P. (2010). Neurologic disease burden in treated HIV/AIDS predicts survival: a population-based study. Neurology.

[bib186] Volkow N.D., Michaelides M., Baler R. (2019). The neuroscience of drug reward and addiction. Physiol. Rev..

[bib187] Wallace D.R. (2006). Estrogen attenuates gp120- and tat1-72-induced oxidative stress and prevents loss of dopamine transporter function. Synapse.

[bib188] Wang S.C., Maher B. (2019). Substance use disorder, intravenous injection, and HIV infection: a review. Cell Transplant..

[bib189] Wang M., Vijayraghavan S., Goldman-Rakic P.S. (2004). Selective D2 receptor actions on the functional circuitry of working memory. Science.

[bib190] Wang J. (2022). Astrocytes in cocaine addiction and beyond. Mol. Psychiatr..

[bib191] Wayman W.N. (2015). Cortical consequences of HIV-1 Tat exposure in rats are enhanced by chronic cocaine. Curr. HIV Res..

[bib192] Wayman W.N. (2016). HIV-1 transgenic rat prefrontal cortex hyper-excitability is enhanced by cocaine self-administration. Neuropsychopharmacology.

[bib193] Wild J.M. (2009). Visual field loss in patients with refractory partial epilepsy treated with vigabatrin: final results from an open-label, observational, multicentre study. CNS Drugs.

[bib194] Woodburn B.M. (2022). Characterization of macrophage-tropic HIV-1 infection of central nervous system cells and the influence of inflammation. J. Virol..

[bib195] Xin W. (2019). Ventral midbrain astrocytes display unique physiological features and sensitivity to dopamine D2 receptor signaling. Neuropsychopharmacology.

[bib196] Xu R. (2012). HIV-1 Tat protein increases the permeability of brain endothelial cells by both inhibiting occludin expression and cleaving occludin via matrix metalloproteinase-9. Brain Res..

[bib197] Xu J.P. (2016). Identification of a small molecule HIV-1 inhibitor that targets the capsid hexamer. Bioorg. Med. Chem. Lett..

[bib198] Xu H. (2016). Persistent simian immunodeficiency virus infection drives differentiation, aberrant accumulation, and latent infection of germinal center follicular T helper cells. J. Virol..

[bib199] Yan Y. (2015). Dopamine controls systemic inflammation through inhibition of NLRP3 inflammasome. Cell.

[bib200] Yang Y. (2010). Cocaine potentiates astrocyte toxicity mediated by human immunodeficiency virus (HIV-1) protein gp120. PLoS One.

[bib201] Yang L. (2016). Role of sigma receptor in cocaine-mediated induction of glial fibrillary acidic protein: implications for HAND. Mol. Neurobiol..

[bib202] Yang H.J. (2022). Elevation of extracellular glutamate by blockade of astrocyte glutamate transporters inhibits cocaine reinforcement in rats via a NMDA-GluN2B receptor mechanism. J. Neurosci..

[bib203] Yu C. (2017). HIV and drug abuse mediate astrocyte senescence in a beta-catenin-dependent manner leading to neuronal toxicity. Aging Cell.

[bib204] Zahm D.S., Brog J.S. (1992). On the significance of subterritories in the "accumbens" part of the rat ventral striatum. Neuroscience.

[bib205] Zheng Y. (2022). Methamphetamine augments HIV-1 gp120 inhibition of synaptic transmission and plasticity in rat hippocampal slices: implications for methamphetamine exacerbation of HIV-associated neurocognitive disorders. Neurobiol. Dis..

[bib206] Zhu J. (2009). HIV-1 Tat protein-induced rapid and reversible decrease in [3H]dopamine uptake: dissociation of [3H]dopamine uptake and [3H]2beta-carbomethoxy-3-beta-(4-fluorophenyl)tropane (WIN 35,428) binding in rat striatal synaptosomes. J. Pharmacol. Exp. Therapeut..

